# Moisture-stable Mg_3_(Bi, Sb)_2_ thermoelectrics enabled by anodic protection

**DOI:** 10.1093/nsr/nwag232

**Published:** 2026-04-17

**Authors:** Xinzhi Wu, Longquan Wang, Gang Wu, Airan Li, Takao Mori

**Affiliations:** Research Center for Materials Nanoarchitectonics (MANA), National Institute for Materials Science (NIMS), Japan; Research Center for Materials Nanoarchitectonics (MANA), National Institute for Materials Science (NIMS), Japan; Research Center for Materials Nanoarchitectonics (MANA), National Institute for Materials Science (NIMS), Japan; Research Center for Materials Nanoarchitectonics (MANA), National Institute for Materials Science (NIMS), Japan; Research Center for Materials Nanoarchitectonics (MANA), National Institute for Materials Science (NIMS), Japan

The conversion between heat and electricity via the thermoelectric effect offers a promising pathway for solid-state cooling [1,2]. Among emerging materials, Mg_3_(Bi, Sb)_2_ has been regarded as a leading candidate to replace conventional Bi_2_Te_3_ owing to its low cost, light weight and excellent performance [3,4]. However, its practical application is critically hindered by its inherent stability issues, particularly its chemical instability under ambient conditions in Bi-rich compositions [5,6].

In atmospheric environments, nanoscale water films inevitably condense on material surfaces, triggering electrochemical reactions. For Mg_3_(Bi, Sb)_2_, its highly negative equilibrium potential (−0.8 to −0.9 V) will induce hydrogen evolution corrosion, producing porous MgO/Mg(OH)_2_ layers that cannot effectively passivate the surface and are continuously stripped by hydrogen bubbles. This issue is further exacerbated in thermoelectric cooling devices, where temperature gradients promote moisture condensation, leading to rapid degradation of thermoelectric performance [5–7].

Previous strategies have mainly relied on external coatings to block H_2_O and O_2_, which have proven to be effective in mitigating degradation [8–10]. In contrast, in a recent work published in *Nature Materials*, Yu *et al*. proposed a different strategy by introducing a sacrificial anodic second phase (Mg_17_Al_12_) into Mg_3_(Bi, Sb)_2_ to achieve cathodic protection (Fig. 1) [7]. The large potential difference between Mg_17_Al_12_ (−1.99 V) and Mg_3_(Bi, Sb)_2_ forms local galvanic couples, where Mg_17_Al_12_ preferentially corrodes and protects the matrix. The dissolution of the anodic phase produces Mg^2^⁺ and Al^3^⁺, which subsequently form dense Mg/Al hydroxide and oxide layers, providing an additional protective barrier.

**Figure 1.**
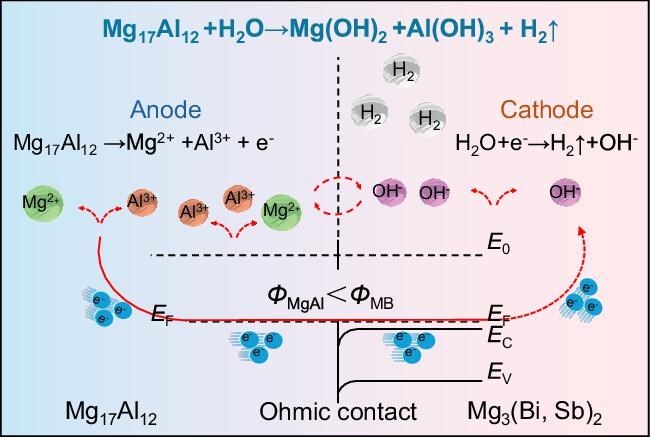
Anodic protection mechanism. Adapted with permission from Ref. [7].

Beyond this classical electrochemical mechanism, the authors further reveal an electronic effect: due to the higher work function of Mg_3_(Bi, Sb)_2_, electrons transfer from Mg₁₇Al₁₂ to the matrix, rendering Mg_3_(Bi, Sb)_2_ negatively charged and thermodynamically less susceptible to oxidation. Notably, the *in situ* formed protective layer exhibits self-healing behavior, ensuring sustained protection.

This dual protection mechanism dramatically improves stability. The corrosion rate is reduced by 92% in air (∼95 μm year⁻^1^) and 86% in water (∼0.36 μm h⁻^1^). At the device level, modules incorporating Mg_17_Al_12_ show comparable performance to commercial Bi_2_Te_3_ at 300 K and superior performance at elevated temperatures, while maintaining stable operation after 28 days under harsh conditions (350 K, 70% relative humidity).

This work shows a paradigm shift from passive barrier protection to active electrochemical design in thermoelectrics. By integrating sacrificial anodic phases, it provides a generalizable strategy to stabilize moisture-sensitive functional materials, as also verified in Mg_2_Sn and CaMg_2_Bi_2_ [7]. Future efforts might focus on optimizing phase distribution, minimizing electronic transport penalties and extending this concept to high-performance but environmentally unstable systems.

## References

[1] Mao J, Liu ZH, Ren ZF. Science 2019; 365: 495–8. 10.1126/science.aax7792 31320557

[2] Chauhan NS, Mori T. Natl Sci Rev 2024; 12: nwae445. 10.1093/nsr/nwae445 39764500 PMC11702843

[3] Wang LQ, Li AR, Wu XZ et al. Natl Sci Rev 2026; 13**:** nwaf507. 10.1093/nsr/nwaf507 41536293 PMC12796818

[4] Liu ZH, Gao WH, Oshima H et al. Nat Commun 2022; 13: 1120. 10.1038/s41467-022-28798-4 35236865 PMC8891317

[5] Li A, Nan P, Wang Y et al. Acta Mater 2022; 239: 118301. 10.1016/j.actamat.2022.118301

[6] Wu XT, Ma XJ, Yao HH et al. ACS Appl Mater Interfaces 2023; 15: 50216–24. 10.1021/acsami.3c12290 37862682

[7] Yu Z, Sun Y, Wu H et al. Nat Mater 2026; doi: 10.1038/s41563-026-02563-0. 10.1038/s41563-026-02563-0 41896460

[8] Shang HJ, Liang ZX, Xu CC et al. Acta Mater 2020; 201: 572–9. 10.1016/j.actamat.2020.10.035

[9] Wu XZ, Lin YJ, Han ZJ et al. Adv Energy Mater 2022; 12: 2203039. 10.1002/aenm.202203039

[10] Ying P, Villoro RB, Bahrami A et al. Adv Funct Mater 2024; 34: 2406473. 10.1002/adfm.202406473

